# Level of non-adherence and associated factors among children on antiretroviral therapy in public hospitals of South Wollo Zone, Northeast Ethiopia

**DOI:** 10.3389/fpubh.2025.1522959

**Published:** 2025-02-28

**Authors:** Endalk Birrie Wondifraw, Fekadeselassie Belege Getaneh, Muluken Amare, Setegn Mihret, Gebeyaw Biset, Birhanu Desu Tefera, Mulusew Zeleke, Fuad Ahmed, Ermias Sisay Chanie

**Affiliations:** ^1^Department of Pediatric and Child Health Nursing, College of Medicine and Health Science, Wollo University, Dessie, Ethiopia; ^2^Department of Emergency and Critical Care Nursing, College of Medicine and Health Science, Wollo University, Dessie, Ethiopia; ^3^Department of Surgical Nursing, College of Medicine and Health Science, Wollo University, Dessie, Ethiopia; ^4^Department of Pediatrics and Child Health Nursing, College of Medicine and Health Science, Debre Berhan University, Debre Berhan, Ethiopia; ^5^Department of Pediatrics and Child Health Nursing, College of Health Science, Debre Tabor University, Debre Tabor, Ethiopia

**Keywords:** non-adherence, antiretroviral therapy (ART), children, South Wollo Zone, Ethiopia

## Abstract

**Background:**

Poor adherence to antiretroviral therapy (ART) occurs when an individual with Human Immune deficiency Virus does not follow the prescribed treatment regimen correctly. This includes missing doses, not taking medication as scheduled, taking medication inconsistently or irregularly, and failing to adhere to specific instructions. The lack of adherence to antiretroviral therapy (ART) among children is a noteworthy issue that necessitates attention. The study aims to determine the level of non-adherence to antiretroviral therapy (ART) and its associated factors in children receiving ART in public hospitals in the South Wollo Zone.

**Methods:**

A multi-center cross-sectional study was conducted among children receiving antiretroviral therapy at South Wollo Zone public hospitals. A single population proportion formula was used to determine the required sample size. A computer-generated simple random sampling method was employed to select the participants. The tools used to assess adherence for all participants were viral load monitoring, Self-reporting, Pill counts, and Pharmacy refill records. Data were collected through face-to-face interviews, and reviewing patients’ documents using a structured checklist. The data were entered into Epi Data version 4.1 and analyzed using STATA 17. Binary logistic regression was employed to evaluate the relationship between the factors and the outcome variable. Variables were considered significant if the *p*-value was less than 0.05.

**Result:**

Of 291 participants, 286 were involved in the study, making the response rate 98.3%. The mean age of the participants was 7.8 years old (±3.64 SD), and half of the 146 children (51%) were male. The overall proportion of ART non-adherence was 24.1% (95% CI: 19.2–29.0%). Positive TB status (Adjusted odd ratio (AOR) = 4.10, 95% CI: 1.90–8.88), diagnostic status not disclosed (AOR = 2.69, 95% CI: 1.43–5.00), and poor caregiver knowledge (AOR = 2.18, 95% CI: 1.04–4.56) were significantly associated with poor adherence.

**Conclusion:**

According to the current study, the level of non-adherence to antiretroviral therapy remains high compared to the targets set by the United Nations Joint Program on HIV/AIDS (UNAIDS) Project 95-95-95. TB co-infection, undisclosed diagnostic status, and poor caregiver knowledge were found to be significantly associated with non-adherence. Before and throughout ART, healthcare providers should provide intense and ongoing counseling to children and their caregivers.

## Background

In 2023, approximately 630,000 individuals died from HIV-related causes, and around 1.3 million people acquired HIV (1). By the end of the year, approximately 1.4 million children aged 0–14 were living with HIV, with 120,000 newly infected. Around 76,000 children succumbed to AIDS-related illnesses. Early testing and treatment are critical to decreasing HIV-related deaths and diseases in this vulnerable group (1). Without timely intervention, half of the children with HIV will die by age 2, and 80% will not survive past their fifth birthday (1–3).

According to an Ethiopian Public Health Institute report published in 2021, around 42,000 Ethiopian children were infected with HIV during the same year. The report also indicates that in 2013 the country’s ART coverage for children was only 12%. Additionally, it highlights that the HIV epidemic among Ethiopian youth has received minimal attention from government initiatives (4, 5).

ART is a combination of antiretroviral medications that can extend and enhance the quality of life of HIV patients by reducing viral loads and raising CD4 cell counts. ART is an HIV medication that should be taken constantly (6). Adherence to appropriate and prolonged treatment is critical to ART success (7).

Poor adherence (non-adherence) to ART occurs when an individual with HIV does not follow the prescribed treatment regimen correctly. This includes missing doses, not taking medication as scheduled, taking medication inconsistently or irregularly, failing to adhere to specific instructions such as taking the drug with food or at designated times, completely discontinuing medication, or not refilling prescriptions as required. Such poor adherence can result in less effective treatment, including treatment failure, higher viral loads, development of drug resistance, and disease progression. Contributing factors may include side effects, the complexity of the regimen, lack of support, or various personal and systemic issues (1, 8, 9).

The study shows that non-adherence levels in Ethiopia ranged from 4.5% at Tikur Anbessa Hospital (10) to 65.5% at Debre Birhan Hospital (11).

Some studies state factors that contribute to poor adherence among children. These factors include socio-economic, socio-demographic, and socio-cultural aspects, as well as problems with service delivery, which can have an impact on adherence to pediatric ART (12, 13). High pill burden, poor palatability, long-term toxicity, ART side effects, ART regimens, and medication dosing are additional factors that can affect adherence. Pediatric ART adherence has also been linked to children’s health, length of time on antiretroviral therapy (ART), knowledge of their HIV status, and psychological variables (10–18).

Routine assessment of medication adherence in clinical care should include increasing the frequency of viral load monitoring after starting or changing medications. Additionally, healthcare providers should ask the child/adolescent and caregiver about the number of missed doses over specific periods (e.g., 1, 3, or 7 days) and request details about the medication, such as its name, appearance, dosage, and frequency of intake. Engaging in conversations with the child and caregiver about potential adherence barriers and strategies to overcome them is essential. Pharmacy-based or clinic-based methods can be used to assess on-time medication refills. Both remote and in-person visits should be utilized to support families, observe ART preparation, handling, and administration, and conduct directly observed therapy (DOT) at home. Finally, announced and unannounced pill counts can be performed by asking individuals to bring medications to the clinic, conducting home visits, or referring them to community health nursing (19, 20).

Non-adherence to ART poses a major challenge to achieving optimal health outcomes for children living with HIV, leading to virologic failure, drug resistance, and increased morbidity and mortality. Understanding the extent of non-adherence is essential for improving pediatric HIV treatment. Ethiopia’s sociocultural, economic, and healthcare contexts significantly influence adherence, and identifying these factors can guide the development of targeted locally relevant interventions.

This study aligns with Ethiopia’s national HIV/AIDS strategic plan and global UNAIDS 95–95-95 goals, contributing to viral suppression and the effort to end the pediatric HIV epidemic. By quantifying non-adherence and its associated factors, the research provides critical data to inform policymakers and healthcare providers, enabling better resource allocation, caregiver training, and implementation of evidence-based strategies.

Non-adherence not only leads to treatment failure but also increases healthcare costs due to the need for second-line therapies and hospitalizations. Addressing adherence early can reduce this burden on the healthcare system. Limited data exist on pediatric ART adherence in Ethiopia, particularly in the study area. This research employs innovative methods, including adherence assessment through viral load measurement, which has not been extensively studied before. The findings will fill critical knowledge gaps, advance pediatric HIV care, and support Ethiopia’s fight against HIV/AIDS. This study aimed to determine the level of non-adherence to ART and its associated factors in children receiving ART in public hospitals in the South Wollo Zone.

## Methods

### Study setting, design, and period

An institution-based cross-sectional study was conducted from June 1 to June 30, 2023, among children receiving antiretroviral therapy at South Wollo Zone public hospitals. It was far from Addis Ababa, a distance of 401 km, and from Bahir Dar, a distance of 471 km. It has a total population of 2,518,862 and 499 health posts, 126 health centers, and 14 hospitals.

### Population

All HIV-infected children <15 years of age who started ART at South Wollo Zone Public Hospitals and their primary caregivers served as our source population for this study. Our study population includes all HIV-infected children <15 years of age who started ART at South Wollo Zone Public Hospitals from September 1, 2019, to June 30, 2023, and their primary caregivers.

### Inclusion criteria

The study included children under 15 who had been on ART for at least 1 month and their primary caregivers.

### Exclusion criteria

Participants with incomplete data (charts lacking significant explanatory and outcome variables) were excluded from this study.

### Sample size determination

A single population proportion formula was used to determine the required sample size with the following statistical assumptions: 22% proportion (p) of the level of non-adherence was taken from a study conducted in northwest Ethiopia (16); 5% margin of error; 10% incomplete or inconsistent data; and 95% confidence intervals (CI).


$$\begin{array}{l} n=\frac{{\left(Za/2\right)}^{2}P\left(1-P\right)}{{\left(d\right)}^{2}}\;n=\;\frac{{\left(1.96\right)}^{2}0.22\left(1-0.22\right)}{{\left(0.05\right)}^{2}}=264 \end{array}$$

Where: *n* = the required sample size, Zα/2 = the standard normal variation, p = proportion (0.22) of the level of non-adherence, and d = margin of sampling error (0.05). By considering 10% of the incomplete or inconsistent data, the final sample size of our study was 291.

### Sampling technique and procedure

**Figure 1:**
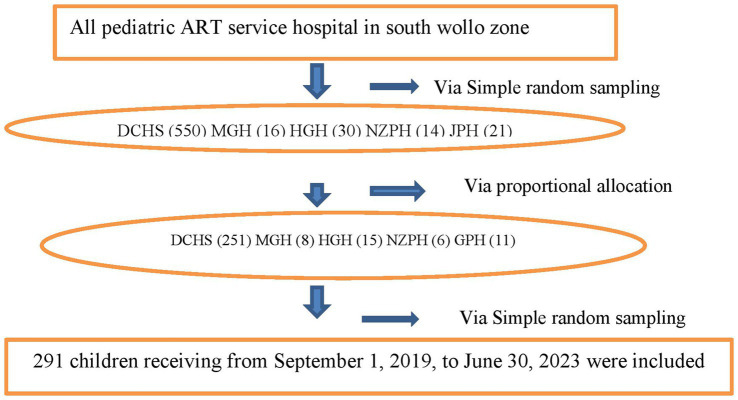
Schematic diagram of sampling procedure among children on antiretroviral therapy at South Woll zone public Hospitals. Dessie Comprehensive Specialized Hospital (DCHS), Hadar 11 General Hospital (HGH), Mekan Selam General Hospital (MGH), Negste Zewditw Primary Hospital (NZPH), and Jamma Primary Hospital (GPH).

From the South Wollo zone hospitals, Dessie Comprehensive Specialized Hospital, Akasta (Hadar 11) General Hospital, Mekan Selam General Hospital, Negste Zewditw Primary Hospital, and Jamma Primary Hospital were selected through a simple random sampling method. In each hospital, the sample size was allocated proportionally. Using the ART registration logbook as the sampling frame, a simple random sampling technique was applied. From September 1, 2019, to June 30, 2023, children on ART were enrolled in the following hospitals: Dessie Comprehensive Specialized Hospital (550 children), Akasta Hadar 11 General Hospital (30 children), Mekan Selam General Hospital (16 children), Negste Zewditu Primary Hospital (14 children), and Jamma Primary Hospital (21 children) (Figure 1).

### Data collection procedure

The data collection checklist was developed using the Federal Ministry of Health’s HIV/ART follow-up and intake records. Data were collected through face-to-face interviews and reviewing patients’ documents using a structured checklist. The data extraction form includes information on socio-demographic variables, treatment and additional drugs, clinical and laboratory data, etc. Under the direction of one MSc nursing practitioner, three BSc nurses with ART service experience and training collected data for 4 weeks in July 2023. Pre-testing was performed on 13 newborns (5% of the sample population) at Dessie Compressive Specialized Hospital before the real data-gathering process.

### Study variables

Dependent variable: Adherence to first-line ART, which was classified as “non-adherent” or “adherent.”

Independent variables: age of the child, sex, residence, Caregiver knowledge, Caregiver marital status, Educational status, HIV disclosure Status, Weight for age, height for age, Hemoglobin (Hgb) level, CD4 counts or %, WHO clinical stages, Duration on ART, CPT (Co-trimoxazole Preventive Therapy), IPT (Isoniazid Preventive Therapy), Treatment failure, and TB status.

### Operational definition

Level of ART adherence: the tools used to assess adherence for all participants were viral load monitoring, Self-reporting, Pill counts, and Pharmacy refill records.

To assess adherence using viral load, first measure the viral load before initiating ART to establish a baseline. This baseline helps in evaluating the treatment’s effectiveness over time. Regular viral load tests, typically conducted every 3–6 months, should be scheduled to track the amount of HIV in the blood. Consistently low viral loads suggest good adherence and effective viral suppression. An undetectable viral load usually indicates that the treatment is working well. However, if the viral load remains low but detectable, it may imply some level of adherence but might also require further evaluation or adjustment of the treatment regimen. Conversely, a high viral load often indicates poor adherence (non-adherence), possible treatment failure, or drug resistance, necessitating a review of adherence strategies and potentially a change in therapy (1, 19, 20).

A 1-Month Self-Recall Report (Visual Analogue Scale): Caregivers were asked to mark on a calibrated line scale from 1 to 10, reflecting how they administered medications to the child over the past month. The marks were converted into percentages to estimate their drug administration adherence for the entire month. Adherence was classified as follows: a mark below 8.5 (<85%) indicated poor adherence, 8.5–9.4 (85–94%) indicated fair adherence, and 9.5 or above (≥95%) indicated good adherence (21, 22).

Pill counts: counting the remaining pills in bottles during routine healthcare visits helps assess adherence (21). Adherence (%) = (Number of pills prescribed - Number of pills returned) × 100.

Pharmacy refill records: pharmacy refill records provide information on when individuals pick up their antiretroviral (ARV) drugs. Irregular pharmacy refill intervals may indicate non-adherence to antiretroviral therapy (ART). Calculation: Proportion of days covered (PDC) = (Number of days the patient had medication) ÷ (Number of days in the observation period) × 100.

Overall adherence measures combining these four adherence measures, an overall response rate of 95% or higher is considered good adherence, 85–94% is fair adherence, and below 85% is poor adherence (non-adherence) (19, 21–23).

Knowledge of caregiver on children ART adherent: caregivers who scored at or above the overall mean on the adherence knowledge assessment were categorized as having good knowledge, while those who scored below the mean were categorized as having poor knowledge (24, 25).

A CD4 count: CD4 below the threshold level was classified based on the age of the child (i.e infants CD4 < 1,500/mm^3^, 12–35 months <750/mm^3^, 36–59 months <350/mm^3^ and ≥ 5 years <200/mm^3^) (26).

Underweight or stunting was defined as weight for age Z-score < −2 SD for under-five children and BMI for age Z-score < −2 SD for older children (26).

Tuberculosis: Cases were detected using sputum or stomach aspirate microscopy, chest X-ray examination, and/or histology, following the Ethiopian Ministry of Health’s TB diagnosis guidelines (27).

ART treatment failure: when an antiretroviral regimen is unable to control HIV infection (21).

The World Health Organization (WHO) clinical staging system: categorizes HIV patients into four stages based on their immune deficiency and clinical symptoms (28).

### Data processing and analysis

The data were entered into Epi Data version 4.1, and STATA 17 was used for the analysis. The table and figures provide an exploration of the descriptive and summary statistics. The outcome level of ART adherence was divided into two categories: “non-adhered” and “adhered.” For each predictor variable, the bivariate logistic regression model was fitted. Furthermore, a multivariate logistic regression model was fitted for those variables with a *p*-value of less than 0.25 in a bivariate analysis. Adjusted odds ratios with 95% confidence intervals and *p*-values were employed to assess the strength of the relationship to determine statistically significant predictors. Variables with a p-value of less than 0.05 in multivariable analysis were regarded as significant predictors of poor levels of adherence. The multicollinearity assumption was assessed using a correlation matrix, confirming that all correlations were below 0.8. Hosmer-Lemeshow test data and the Omnibus assumption were also used to check the model fitness.

### Ethical approval and consent to participate

Approval was obtained from the Research and Ethical Review Committee of Wollo University College of Medicine and Health Sciences, with reference number CMHS/RCPGC/03/14. All hospital administrations and the ART clinic’s focal person provided permission letters. Written informed consent was obtained from caregivers willing to participate in the study. The names or identification numbers of the children were not permitted to be recorded in the data. Furthermore, the study was conducted by the ethical principles of the Declaration of Helsinki.

## Result

### Socio-demographic characteristics of the study participants

Of 291 participants, 286 were involved in the study and five incomplete ones were discarded. As a result, 286 children were included in the analysis, yielding a completion rate of 98.3%. The mean age of the participants was 7.8 years old (±3.64 SD), and half of the 146 (51%) children were male. More than half of the 176 (61.5%) children knew that they had HIV, and the vast majority (83.6%) lived in cities. Almost half of the 156 (54.5%) caregivers had a good understanding of the importance of ART (Table 1).

**Table 1:** Socio-demographic characteristics of children on antiretroviral therapy at South Wollo Zone Public hospitals, 2024.

Characteristics	Categories	Frequency	Percentage
Age of the children (years)	<5 years	47	16.4%
	5–9 years	36	12.6%
	≥10 years	203	71%
Sex	Male	146	51%
	Female	140	49%
Residence	Urban	37	13%
	Rural	249	87%
HIV disclosure status	Yes	176	61.5%
	No	110	38.5%
Knowledge of the caregiver about ART drugs	Good	156	54.5%
	Poor	130	45.5%

### Clinical and treatment-related characteristics of children on ART

Approximately half of the 175 children (55%) had a CD4 count or percentage above the threshold. Sixty-one percent (61%) of the participants were in WHO stages I and II, while 16.8% had TB. Regarding ART duration, 252 participants (88.1%) had been on ART for more than 34 months. Additionally, 111 participants (39%) had anemia (hemoglobin <10 mg/dL). During data collection, viral load tests were conducted for 217 children (75.8%) within the past 12 months. Of these, 59 children (20.7%) had a high viral load (≥1,000 copies/mL) (Table 2).

**Table 2:** Clinical and treatment-related characteristics of children on antiretroviral therapy at South Wollo Zone Public hospitals, 2024.

Characteristics	Categories	Frequency	Percentage %
Weight for age	Normal	249	87%
	Underweight	37	13%
Height for age	Normal	251	88%
	Stunting	35	12%
CD4 counts or % level	Below threshold	129	45%
	Above threshold	157	55%
WHO clinical staging	I/II	174	61%
	III/IV	112	39%
Levels of HGB	> 10 mg/dL	111	39%
	≤10 mg/dL	175	61%
IP	Given	205	72%
	Not given	81	28%
CPT	Given	154	54%
	Not given	132	46%
TB status positive	Yes	48	16.8%
	No	238	28%
Viral RNA copies per ml within 12 months	≥1,000 mL	59	20.7%
	<1,000 mL	200	69.9%
	Not done	27	9.4%
Duration on ART	<34 months	34	11.9%
	>34 months	252	88.1%

Hemoglobin (Hgb) level, CPT (Co-trimoxazole Preventive Therapy), IPT (Isoniazid Preventive Therapy), antiretroviral therapy (ART), and Tuberculosis (TB).

### Level of ART non-adherence

In this study, the overall proportion of ART non-adherence was 24.1% (95% CI: 19.2–29.0%) of the study participants.

### Factors associated with poor adherence to ART

Bivariate and multivariate logistic regressions were used to fit all variables that met the chi-square assumption. In the bivariable logistic regression analysis, age of the child, sex, residence, knowledge of the caregiver, HIV disclosure status, weight for age, height for age, HGB level, CD4 count or percentage, WHO stage, CPT user, IPT user, TB status, and duration of ART were each given a *p*-value of less than 0.25 and fitted into a multivariable logistic regression model. Positive TB status, diagnostic status not disclosure, and poor caregiver knowledge were found to be significantly associated with poor adherence at the multivariate level, with *p*-values of less than 0.05 (Table 3).

**Table 3:** Bivariate and multivariate analyses of factors associated with poor adherence to ART at South Wollo Zone Public Hospitals (*n* = 286).

Variable	Category	Level of adherence		COR, 95% CI	*p*-value	AOR, 95% CI	*p*-value
		Good	Poor				
Age of the children (years)	<5 years	35	12	1.13 (0.54–2.36)	0.72	–	
	5–9 years	26	10	1.27 (0.57–2.83)	0.54	–	
	≥10 years	156	47	1	–	–	
Sex	Male	112	34	1	–		
	Female	105	35	0.91 (0.53–1.56)	0.73	–	
Residence	Urban	23	14	1	–	1	
	Rural	194	55	2.14 (1.03–4.45)	0.04	1.77 (0.75–4.17)	0.21
Knowledge of the caregiver about ART drugs	Good	134	22	1	–	1	–
	Poor	83	47	3.44 (1.94–6.13)	0.00	**2.18 (1.04–4.56)**	**0.03**
HIV disclosure status	Yes	150	26	1	-	1	-
	No	67	43	3.70 (2.10–6.51)	0.00	**2.69 (1.43–5.00)**	**0.00**
Weight for age	Normal	195	54	1	–	1	–
	Underweight	22	14	2.46 (1.19–5.06)	0.01	1.34 (0.57–3.14)	0.49
Height for age	Normal	197	54	1	–	1	
	Stunting	20	15	2.76 (1.31–5.70)	0.00	1.30 (0.53–3.18)	0.55
CD4 counts or % level	Below threshold	96	33	1.15 (0.67–1.98)	0.60	–	
	Above threshold	121	36	1	–	–	
WHO clinical staging	I/II	138	36	1	–	1	–
	III/IV	79	33	1.60 (0.92–2.76)	0.09	1.07 (0.52–2.18)	0.16
Levels of HGB	> 10 mg/dL	135	40	1	–	–	
	≤10 mg/dL	82	29	1.19 (0.68–2.07)	0.52	–	
IP	Given	156	49	1	–	–	
	Not given	61	20	1.04 (0.57–1.89)	0.88	–	
CPT	Given	115	39	1	–	–	
	Not given	102	30	0.86 (0.50–1.49)	0.69	–	
TB status positive	Yes	19	29	7.55 (3.86–14.77)	0.00	**4.10 (1.90–8.88)**	**0.00**
	No	198	40	1	–	1	–
Duration on ART	<34 months	22	12	1	–	1	–
	>34 months	195	57	0.53 (0.25–1.14)	0.10	1.65 (0.65–4.19)	0.30

Bold values indicate statistically significant results (*p* < 0.05).

## Discussion

Adherence is crucial for the success of antiretroviral therapy (ART), yet non-adherence remains a significant challenge, particularly among children receiving ART. This cross-sectional study aimed to assess the level of non-adherence to ART in children with HIV and identify the factors contributing to it.

According to our findings, the total proportion of ART non-adherence among the study participants was 24.1% (95% CI: 19.2 –29.0%). Furthermore, TB co-infection, HIV non-disclosure status, and poor caregiver knowledge were significantly related to poor adherence. This finding is consistent with research conducted in public hospitals in the northeast (21.4%) (11) and northwest (21.1%) (16) Ethiopia, Uganda (21%) (29), Nigeria (23.9%) (30), and Myanmar (23.8%) (31). However, this percentage is less than that of studies conducted in Jimma (36.2%) (32), Fiche Hospital (36%) (33), and Tikur Anbessa Hospital (65.2%) (10). On the other hand, this result is higher than that of research conducted in Ethiopia’s Tigray region (15.2%) (25). This disparity could be explained by the differences in ART-adherence diagnostic techniques. Furthermore, inaccurate reporting is more common in low-income nations than in middle-income countries due to a shortage of skilled healthcare providers and caretakers. This variation considered socioeconomic level, study design, adherence measurement methods, sample size, and setting differences. This variation may reflect differences in local healthcare systems, patient demographics, or methodologies. Higher adherence rates in some studies could be due to better support systems, more effective adherence interventions, or differing standards of care. Conversely, the lower adherence rate could be linked to unique regional challenges or disparities in healthcare resources and support. These differences highlight the need for context-specific strategies to address ART adherence and improve treatment outcomes across diverse settings.

This study result indicates that caregivers with poor knowledge about ART (antiretroviral therapy) drugs are 2.18 times more likely to experience non-adherence compared to those with better knowledge [AOR (: 95% CI) = 2.18 (1.04–4.56)]. This finding is consistent with the results of studies in the Oromia region (24), northeast Ethiopia (34), northern Ethiopia (25) and India (35). This result can be justified by the crucial role caregivers play in managing and supporting ART adherence. Caregivers with limited knowledge may struggle to understand the importance of adherence, proper medication administration, and potential side effects, which can lead to inconsistent or incorrect use of ART. This lack of knowledge can directly impact the effectiveness of the treatment and the patient’s overall health, thereby increasing the likelihood of non-adherence. Improving caregivers’ knowledge about ART is essential for enhancing treatment adherence. Caregivers with a better understanding of ART are more likely to support proper medication use and adherence, which can lead to better health outcomes for the children. Therefore, targeted education and training programs for caregivers should be a priority to reduce non-adherence and improve the effectiveness of ART.

In this study, the odds of non-adherence among children with TB/HIV co-infection were more than four times higher than those in children without TB co-infection [AOR = 4.10, 95% CI (1.90–8.88)]. This finding is similar to those of investigations conducted in northwest Ethiopia (16), Nigeria (30), and Peru (36). The reason for this occurrence could be attributed to the administration of medication for the confection, therapy, and antiretroviral therapy in combination, which may lead to an increase in the number of pills required to be taken, interactions between drugs, and an increasing rise in the occurrence of unfavorable drug reactions. These factors collectively can hinder adherence to antiretroviral drugs (16). The increased risk of non-adherence in children with TB/HIV co-infection underscores the need for specialized support and management strategies. The interaction between treatments and the added burden of co-infection can significantly affect adherence. Targeted interventions and integrated care are crucial for improving adherence and overall treatment outcomes in these children.

This study found that children unaware of their HIV status (AOR = 2.69, 95% CI: 1.43–5.00) were more likely to exhibit poor adherence compared to those who were aware of their status. Similar findings have been reported in other studies (17, 18, 37, 38). Children who do not know their HIV status may lack understanding of the importance of their treatment regimen, which can result in lower motivation to adhere to prescribed ART. Awareness of their condition often encourages better adherence as it fosters a clearer understanding of the necessity of consistent medication for their health. This result aligns with similar studies, reinforcing the need for HIV status disclosure and education to improve adherence outcomes in pediatric patients (39). This result implies that HIV status disclosure is crucial for improving ART adherence in children.

Limitations of the study were we did not account for variables related to household dynamics, such as the quality of the caregiver-child relationship, instances of violence and maltreatment, or health system-related factors. Additionally, factors such as the mode of HIV transmission (vertical or acquired), alcohol abuse, depression, caregiver and patient education levels, the caregiver-patient relationship, age at first diagnosis, and parental HIV status were not considered, which may have influenced the results. Another limitation is that caregivers may inaccurately recall adherence behaviors or knowledge. Since the study was conducted in a specific region, the findings may not apply to other areas with differing healthcare systems, resources, or cultural contexts.

## Conclusion and recommendation

According to the current study, the level of non-adherence to antiretroviral therapy remains high compared to the targets set by the United Nations Joint Program on HIV/AIDS (UNAIDS) Project 95–95-95. TB status-positive (co-infection), diagnostic status not disclosed, and poor caregiver knowledge were discovered to be significantly related to poor adherence. Implement targeted education programs to improve caregiver knowledge about ART and its importance. Develop strategies to encourage and support the disclosure of diagnostic status to ensure better adherence. Strengthen integrated care approaches for children with TB/HIV co-infection to address the unique challenges they face in adhering to treatment. These measures can help improve adherence rates and overall health outcomes for affected children.

## Data Availability

The raw data supporting the conclusions of this article will be made available by the authors, without undue reservation.
